# Psychosocial distress in young adults surviving hematological malignancies: a pilot study

**DOI:** 10.1007/s00432-022-04527-8

**Published:** 2022-12-17

**Authors:** Andreas Wittwer, Kristin Sponholz, Jochen J. Frietsch, Paul Linke, Peter Kropp, Andreas Hochhaus, Inken Hilgendorf

**Affiliations:** 1 Abteilung für Hämatologie und Internistische Onkologie, Klinik für Innere Medizin II, Universitätsklinikum Jena, Am Klinikum 1, 07747 Jena, Germany; 2 Psychotherapie und Psychoonkologie, Institut für Psychosoziale Medizin, Universitätsklinikum Jena, Jena, Germany; 3 Medizinische Klinik und Poliklinik II, Universitätsklinikum Würzburg, Würzburg, Germany; 4 Institut für Medizinische Psychologie und Medizinische Soziologie, Universitätsmedizin Rostock, Rostock, Germany

**Keywords:** Adolescents and young adults (AYA), Cancer survivor, Psychosocial distress, Quality of life, Sequelae

## Abstract

**Purpose:**

Survivors of cancer during young adulthood face multiple psychosocial challenges following treatment. This study explores psychosocial distress and unmet needs among young adult survivors treated of hematological malignancies.

**Methods:**

A total of 85 young adults aged between 18 and 39 years at time of diagnosis, were invited to join the survey after the completion of treatment with curative intent. Sociodemographic data and the need for advice were gathered with a self-report questionnaire. A set of standardized questionnaires for quality of life (EORTC QLQ-C30), psychosocial stressors (PHQ-S), fear of progression (PA-F-KF), cancer-related fatigue (EORTC QLQ-FA12), and symptoms of anxiety (GAD-7) or depression (PHQ-9) was employed. Descriptive statistics and multivariate analysis were conducted.

**Results:**

Forty-seven young adult cancer survivors responded. A quarter of patients (26%) reported depressive symptoms, 15% suffered from anxiety, 36% from fear of progression, and 21% reported increased psychosocial stressors. They had a lower QoL than the general population and reported poorer outcomes on all single-item and multi-symptom scales. Employment was significantly associated with lower levels of psychosocial distress, anxiety, fatigue, and better QoL.

**Conclusion:**

Young adult cancer survivors exhibited a high disposition for psychosocial distress. They reported excessive demands in everyday life and resumption of work. However, a longitudinal study of young adult cancer survivors is needed to confirm the results of this pilot study. In future, psycho-oncological and social support need to become an inherent part of the aftercare of survivors of young adult cancer survivors.

**Supplementary Information:**

The online version contains supplementary material available at 10.1007/s00432-022-04527-8.

## Introduction

In Germany, 16,500 young adults aged from 18 to 39 years are diagnosed with cancer each year (Hilgendorf et al. 2021). Even though the overall survival rates of young adults with cancer have steadily improved, the disease and its treatment are often the cause of several serious delayed consequences including effects on psychosocial health and financial constraints.

The years that mark the transition from childhood to adulthood come with their own set of complex challenges. A cancer diagnosis in this critical period presents an unfathomable set of new hurdles. The loss of autonomy and independence may contribute to later psychosocial problems. Adolescents and young adult (AYA) cancer survivors complain about difficulties with reintegration into education or work. More than 50% of full-time workers or students reported problems in this area after diagnosis (Parsons et al. 2012). Survivors of cancer during young adulthood struggle with restricted physical and mental work capacity and increased periods of sick leave (Ketterl et al. 2019). They suffer from cancer-related cognitive dysfunction and reported problems with attention, memory, and processing speed (Husson et al. 2017b; Prasad et al. 2015; Vetsch et al. 2018). As a consequence, more than half of young adults reported a reduced ability to work after cancer treatment (Brock et al. 2021). Thus, cancer can have a negative impact on the career development of survivors and lead to financial burden and distress (Guy et al. 2014; Leuteritz et al. 2021). In addition, the emotional and financial strains of cancer may lead to marital stress for younger cancer survivors, since they were more likely to divorce or separate from their spouse than their age-controlled counterparts (Kirchhoff et al. 2012).

While several international reports are available on the topic of long-term survivorship after tumor therapy in young adulthood, there is a lack of studies dealing with young adults specifically in Germany (Richter et al. 2015). In addition, there is no broadly accessible needs assessment or established risk stratification system available (Hilgendorf et al. 2021) and orientation guides for long-term survivors as well as new and innovative survivorship programs need to be developed (Bergelt et al. 2022).

The purpose of the current pilot study was to investigate the experiences and psychosocial needs of young adults after treatment of a hematological malignancy. Furthermore, the impact of employment on psychosocial issues was explored to optimize our provision of information and to create needs-based and structured interventions.

## Patients and methods

### Patients

A monocentric survey was conducted among 85 young adults aged between 18 and 39 years at the time of diagnosis of hematological malignancy (acute leukemia, lymphoma, myelodysplastic syndrome, or multiple myeloma). These patients had completed medical treatment with curative intent between January 2010 and October 2017 at the Jena University Hospital, Germany. Local ethics committee approved the study (5234-07/17) and all analyses were in line with the Declaration of Helsinki.

Suitable patients were identified by searching the clinic’s database. They had to be in regular follow-up without evidence of relapse. Survivors who moved elsewhere or attended follow-up in an outpatient setting were still invited to participate as long as they had attended at least one aftercare appointment at the Jena University hospital. Patients were excluded from study participation if they did not provide written consent or were unable to speak German. There was a cover letter explaining the aims of the study and a stamped addressed return envelope. All letters were delivered by post, and six letters were returned to sender. Two months after the invitation to participate in the study, 52 patients received a reminder letter with another copy of the questionnaire.

### Instruments

Sociodemographic data were gathered with a self-report questionnaire. The form included information on gender, age, height, weight, employment, marital status, and parenthood. In addition, data about diagnosis, treatment, and disease timeline were collected from the electronic health record.

QoL was measured by the European Organization for the Research and Treatment of Cancer Quality and Life Questionnaire-Core 30 (EORTC QLQ-C30) (Aaronson et al. 1993). It contains one multi-item QoL-scale, five multi-item function scales (physical, role, emotional, cognitive, social), six single-item (dyspnea, insomnia, appetite loss, constipation, diarrhea, financial difficulties), and three multi-item (nausea, pain, fatigue) symptom scales. The results of young cancer patients were compared with the published results of healthy controls used as an age-matched reference population consisting of 585 individuals (Geue et al. 2014). Cancer-related fatigue (CRF) was measured with the EORTC QLQ-FA12 (Weis et al. 2017). This additional module of the EORTC QLQ-C30 measures the interference of physical, emotional, and cognitive fatigue on daily activities and social life. The feasibility of this questionnaire in young adults with cancer had been reported (Friedrich et al. 2018). The guidelines provided by Cocks et al. were used for interpretation of point differences between two results on a single scale (Cocks et al. 2011). For every subscale, there is an individual classification in trivial, small, medium, and large differences with the exception of emotional function. In addition, participants completed self-reported measures assessing the fear of cancer recurrence and disease progression (PA-F-KF) (Hinz et al. 2015; Mehnert et al. 2006). Subscales of the Patient Health Questionnaire (PHQ-D) (Spitzer 2001) were used to analyze symptoms of anxiety (Generalized Anxiety Disorder Scale-7, GAD-7) (Löwe et al. 2008; Spitzer et al. 2006), of depression (PHQ-9) (Löwe et al. 2004), and of stress (PHQ-S).

A modified version of the previously published questionnaire from our group (Pulewka et al. 2021) was used to evaluate the need for advice, to measure difficulties in daily routine or return to work. Respondents were asked to rate on a one-to-six graded Likert scale (not a concern, very small, small, moderate, severe, and very severe) how much of a concern the item was.

### Statistical analysis

To maintain anonymity, consent forms were separated and the questionnaires labeled with numbers. Further analyses and results were not linked to names or personal identifiers. Statistical analyses was conducted using SPSS (version 25, IBM). Descriptive statistics based on the available data were reported, stating absolute and percentage frequencies for categorical data and mean and standard deviation (SD) for continuous variables (EORTC QLQ-C30, QLQ-FA12, PA-F-KF, informational needs). For normal distributed variables, *T* tests were employed to analyze group differences (QLQ-C30, QLQ-FA12, PA-F-KF). Multiple linear regression analysis was used to identify factors associated with psychological distress and QoL. The dependent variables in these regression models were QoL (QLQ-C30), fatigue (EORTC QLQ-FA12), depression (PHQ-9), anxiety (GAD-7), and fear of cancer recurrence (PA-F-KF). The following sociodemographic and medical parameters were defined as independent variables for each model: gender, body mass index, age at the time of the survey, relationship status, number of children, level of education, employment status, diagnosis (leukemia or other diagnosis), and treatment (received an SCT or not). Some of the variables have been transformed to dichotomous data to proceed the calculation. Significance of the models was analyzed by the F test. A *p* value of < 0.05 was considered significant.

## Results

### Sample characteristics

Eighty-five patients were invited to participate in the pilot study. The data from 47 (55%) respondents were available. Six patients had left no forwarding address. One person declined to participate and the remaining patients did not respond. The demographic and clinical characteristics of the participants are presented in Table 1. Their median age when surveyed was 35 (SD 5.86) years. The median time since diagnosis was 54 months (with a range of 10–96 months). The majority were female (*n* = 27; 57%) and lived in a stable relationship (*n* = 28; 60%). The body mass index (BMI) exceeded 30 kg/m^2^ in 12 of 47 (26%) participants and of those the BMI was 30–35 kg/m^2^ (obesity class I) in 9 patients, 35–40 kg/m^2^ (obesity class II) in 1 patient and over 40 kg/m^2^ (obesity class III) in 2 participants, respectively. Most of the young adults were employed (*n* = 37; 79%), including one student and three trainees. Ten of the study participants were unemployed. Eight of these were incapable of working due to disability.

**Table 1:** Description of young adults sample (*n* = 47)

Characteristics	*n*	%
**Gender**		
Male	20	43
Female	27	57
**Age (at the time of the survey, years)**		
18–25 years	4	9
26–32 years	10	21
33–39 years	19	40
> 40 years	10	30
**Body mass index (BMI, kg/m**^2^**)**		
< 18.5	1	2
18.5–25	18	38
25–30	15	32
> 30	12	26
Prefer not to answer	1	2
**Graduation**		
No graduation	1	2
Lower secondary graduation	5	11
Ordinary level	17	36
High school	11	23
Academic degree	12	26
Prefer not to answer	1	2
**Marital status**		
Single	19	40
Married/partnered	28	60
**Children**		
Yes	21	45
No	26	55
**Disease**		
Acute leukemia or myelodysplastic syndrome	16	34
Hodgkin lymphoma	10	21
Non-Hodgkin lymphoma	17	36
Multiple myeloma	4	9
**Treatment**		
Chemotherapy only	19	40
Stem cell transplantation after chemotherapy	18	38
Combination of chemotherapy and radiation	10	21
**Employment**		
Employed (including students and trainees)	37	79
Unemployed	10	21
**Year of diagnosis**		
2010	3	6%
2011	3	6%
2012	8	17%
2013	11	23%
2014	3	6%
2015	10	21%
2016	7	15%
2017	2	4%

### Quality of life

**Fig. 1:**
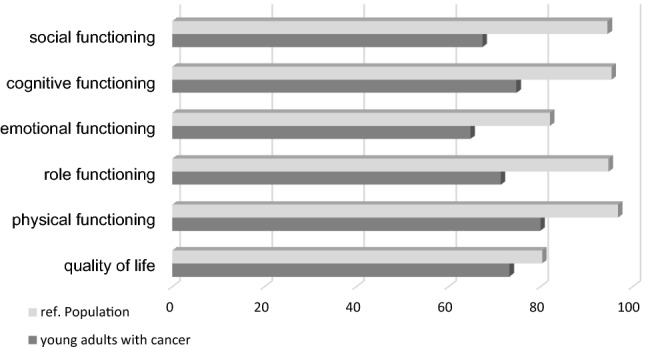
Functioning and quality-of-life scores of the QLQ-C30. Comparison of the study population and the reference population (Geue et al. 2014)

The mean value of the global QoL in young adult cancer survivors on the EORTC QLQ-C30 was 73.3 (SD 11.7) points. This is significantly lower than in the general population [80.4 point (SD 18.3, *T* score − 2.587, *p* = 0.013)] points. The full results of the EORTC QLQ-C30 are presented in the supplementary material in Table S1. Male patients reported a significantly lower QoL (69.4, SD 20.6, point difference to the male general population of 13.3, *T* score − 2.654, *p* = 0.016) than female patients (75.9, SD 15.4, point difference to the female general population 3.3, *T* score − 1.06, *p* = 0.297), respectively. With regards to physical, role, emotional, cognitive, and social function, the mean value of each scale was significant lower (*p* < 0.001) in cancer survivors than in the reference group (Fig. 1). There was a medium difference for physical and role function and a large difference for cognitive and social function, according to the classification (Cocks et al. 2011). Point differences are shown in Fig. 1. In addition, young adult cancer survivors reported significantly poorer outcomes on all single-item and multi-symptom scales. The largest point differences were observed for insomnia (29.0) and financial difficulties (19.7) on single-item symptom scales and for fatigue (27.5) and pain (19.1) on multi-symptom scales. Gender had no significance for the results of the function scales, quality of life, nor the other scales. Only for dyspnea, there was a significant difference between male and female patients of the study population. Employment after cancer treatment was associated with better QoL. None of the other included variables, e.g., gender, graduation, marital status, and having children, contributed significantly to the variance in the regression analysis.

### Fatigue

The overall fatigue score (EORTC QLQ-FA12) is composed of three components: physical, cognitive, and emotional fatigue. The mean score reported here was 28.2 (male: mean 29.8, SD 25.6 female: mean 27.0, SD 28.2, *p* = 0.734). The reported fatigue between male and female young cancer survivors was comparable on all three scales of the EORTC QLQ-FA12. Physical (mean 40.7, SD 30.1, point difference 4.2, *p* = 0.662) and cognitive fatigue tended to be higher in male cancer survivors (mean 17.6, SD 24.5, point difference 4.0, *p* = 0.602), whereas woman divulged more emotional fatigue (mean 20.2, SD 31.3, point difference 0.3, *p* = 0.974) than male patients. In addition, the extent to which fatigue interfered with activities of daily life and social life had a mean value of 31.2 (SD 31.0) and 21.7 (SD 30.8) points, respectively, across the whole group. The impact of tiredness on activities of daily life and social life did not differ significantly between the genders. Regular employment after cancer treatment was associated with lower fatigue scores (Table 2). None of the other variables studied, e.g., sex, graduation, marital status, and parenthood, contributed significantly to the explained variance in the regression analysis.

**Table 2:** Multiple regressions models

	Dependent variable:						
	Stress	Anxiety	Depression	QoL	Fatigue	Fear of cancer recurrence	
Unemployed	0.369*	0.422*	0.377*	− 0.635***	0.539**		
Educational Qualifications Children						0.407* − 0314	
Observations	45	45	45	44	44	44	
Adjusted *R*^2^	0.21	0.206	0.174	0.259	0.226	0.418	
*F* statistic	2.300*	2.269*	2.033	2.668*	2.397*	4.509***	

Strength of prediction given by beta coefficients, note: *p < 0.05; **p < 0.01; ***p < 0.001

### Psychosocial distress, anxiety, and depression

Ten (21%) patients reported increased psychosocial stressors. The most frequent stressors were “*worries about health”* and “*worries about weight or appearance”* affecting 23% of patients, respectively. The second most severe stressor was *“little or absent libido”* (21%), 15% were bothered a lot by “*financial problems”*, 13% by *“stress at work or at school*”, and 11% by *“relationship difficulties”.*

Seven (15%) patients suffered from moderate or severe symptoms of anxiety and 12 (26%) patients from moderate or severe symptoms of depression. The results of the questionaries are depicted in Table 3. Regular employment after cancer treatment was associated with lower levels of psychosocial distress, anxiety, and depression (Table 2). However, the regression model did not show a significant difference in depressive symptoms in relation to employment status (*p* = 0.065). None of the other included variables, e.g., sex, graduation, marital status, and parenthood, contributed significantly to the explained variance in the regression analysis.

**Table 3:** Results for the presence of depression (PHQ-9 questionnaire) or anxiety (GAD-7 questionnaire)

		Depression	Anxiety
Mean (standard deviation)		5,77 (5,63)	4,33 (4,66)
Minimal (< 5)		26 (55,3%)	30 (63,8%)
Mild (5–9)		9 (19,1%)	10 (21,3%)
Moderate (> 9)		8 (17,0%)	5 (10,6%)
Severe (≥ 15)		4 (8,5%)	2 (4,3%)

### Fear of cancer recurrence

Seventeen (36%) patients experienced a high level of fear of cancer recurrence. Young cancer survivors having children were more likely to report a higher fear of cancer recurrence than those without (beta = − 0.314, *p* = 0.035, adjusted *R*^*2*^ = 0.418). In contrast, survivors with a higher educational level reported less fear of cancer recurrence (Table 2) No association between age, sex, marital status, time since treatment, and fear of cancer recurrence was found. However, patients using the internet as a source of information (36/46, 78%) reported higher values of fear of cancer recurrence (mean 33.7, SD 10.15) than those who did not (mean 25.3, SD 8.76; *p* = 0.022).

### Informational needs

All participants were asked to rate their personal need for advice, ranging from very low to very intensive.

#### Medical issues

The medical consultation time was sufficient for three-quarters (75%) of patients who responded. As a source of additional information, the majority of young adults with cancer used the Internet (78%), 57% talked to other patients, and 52% used information brochures. Seven patients (15%) used information found in web forums and six patients (13%) used the *Young Cancer Portal* (www.junges-krebsportal.de/) for online counseling.

Aftercare (mean 5.59, SD 0.80), sequelae (mean 5.50, SD 0.78) and medication (mean 5.28, SD 0.93) were the most important topics that patients required further information on. These were followed by infertility (mean 4.87, SD 1.44), nutrition (mean 4.7, SD 1.09), skin changes/rash/alopecia (mean 4.42, SD 1.18), and physical activity and exercise (mean 4.11, SD 1.16). Advice on alternative medicine (mean 3.67, SD 1.68) was of lower importance.

#### Psychological issues

Approximately a third of the patients either accepted (34%) or refused (34%) the offer of psycho-oncological support during therapy. The most important rated topics with regards to the need for advice included coping strategies (mean 5.07, SD 1.22), family, relationship and friends (mean 4.70, SD 1.36), fatigue (mean 4.28, SD 1.53), and body shape/sexuality (mean 4.04, SD 1.35). Information regarding relaxation techniques (mean 3.70, SD 1.46) was less important.

#### Social issues

Issues regarding rehabilitation (mean 4.78, SD 1.51) and early retirement (mean 4.35, SD 1.66) were of high interest, followed by information on acquiring a level of care (mean 3.7, SD 1.70).

## Discussion

Young adults who receive the potentially life-threatening diagnosis of cancer at a crucial stage of their development and transition to adulthood are particularly vulnerable to psychosocial problems. The aim of this pilot study was to analyze the experiences and psychosocial needs in young adult patients after the treatment of a hematological malignancy.

AYA cancer survivors complain frequently about a reduced health-related QoL in comparison to healthy peers (Husson et al. 2017a; Murnane et al. 2021; Quinn et al. 2015; Salsman et al. 2014; Smith et al. 2013). In the present study, male patients reported a poorer QoL compared to female patients and the healthy controls. This finding conflicts with data published by Geue and colleagues (Geue et al. 2014). However, they included a more heterogenous group of young adult cancer survivors with a high number of woman with solid tumors, e.g., 53.25% women with breast cancer. In the present study, we included only patients with hematological malignancies, with 38% of those being HSCT recipients. In line with our results, a poorer QoL in male cancer patients in comparison with age- and sex-matched controls was also found in a cross-sectional study. This was more pronounced in cancer survivors with androgen deficiency (Greenfield et al. 2010). Testosterone levels were below the lower level of normal in 84% of male recipients of HSCT for acute leukemia (Vaezi et al. 2016). Hypogonadism appears to be common after HSCT (Inamoto and Lee 2017) and may be another explanation for the lower QoL and higher physical fatigue levels (Savani et al. 2011) in males compared to females within our study.

Patients who underwent very intensive cancer treatment were four times more likely to believe that cancer had a negative impact on their plans for school or work compared to those receiving less-intensive treatment (Parsons et al. 2012). This study found that being unemployed is associated with lower levels of health-related QoL, which is consistent with the findings of other studies (Husson et al. 2017b; Warner et al. 2016). Compared with healthy controls, AYA cancer survivors reported significantly higher levels of concern regarding retirement (25.5 vs 16.9%), standard of living (20.4 vs 12.9%), monthly bills (14.9 vs 10.3%), and housing costs (13.6 vs 8.9%) (Zheng et al. 2020). In addition, 57.6% of them reported severe/moderate financial worry intensity and 27.0% experienced severe/moderate food insecurity intensity (Zheng et al. 2020). Sisk and colleagues found that AYA with hematological malignancies were less likely to return to their pre-cancer working status after their treatment (Sisk et al. 2020). The complete financial independence of AYA cancer patients decreased from 37%, 6 months prior diagnosis, to 25% at 4 months and 30% at 12 months post-diagnosis (Sisk et al. 2020). It is tempting to suggest that financial distress may promote the additional finding of insomnia among young cancer survivors when compared to their healthy peers. However, trouble sleeping was found to be associated with greater mental distress among AYA cancer survivors (Kaul et al. 2017) and a recent study showed that insomnia correlated strongly with fatigue in breast cancer survivors (Haque et al. 2021).

AYA cancer survivors are at increased risk of developing fatigue in comparison with population-based controls (Murnane et al. 2021; Poort et al. 2017). A BMI above 25 kg/m^2^ in cancer survivors is associated with clinically significant cancer-related fatigue (Gerber et al. 2011; Meeske et al. 2007). In addition, the severity of fatigue symptoms is associated with higher BMI (Meeske et al. 2007). AYA, particularly those with ALL or lymphoma, are at risk of being overweight or obese during their cancer survivorship (van der Haak et al. 2021). In the present study, nearly a quarter of young adults who survived a hematological malignancy had a BMI above 30 kg/m^2^. This is of importance as obesity is an independent risk factor for cardiovascular disease and many young adults have also had cardiotoxic chemotherapy. Data from Kaiser Permanente AYA Cancer Survivors Study showed that survivors are at increased risk for developing cardiovascular disease (Chao et al. 2016). Although this finding may have been due to the cardiotoxic side effects of chemotherapeutic agents and irradiation, the monitoring of additional cardiovascular risk factors, such as obesity and atherogenic dyslipidemia, is an important issue in the aftercare of young cancer survivors. In addition, the promotion of regular physical activity may help to reduce the burden of fatigue as the positive effects of exercise on fatigue, insomnia, and other health outcomes are well known (Hauken et al. 2014; Schumacher 2018).

Poort et al. reported a strong correlation between fatigue severity and psychological distress in AYA with cancer (Poort et al. 2017). In addition, AYA cancer survivors reported moderate and severe mental distress (23.2 and 8.4%) significantly more often than a cancer-free comparison group (16.9 and 3.0%, *p* < 0.001) (Kaul et al. 2017). In the present study, 21% of participants experienced elevated levels of distress, with “*worries about health”,* “*worries about weight or appearance”,* and *“little or absent libido”* as most the frequent stressors. In addition, their most important rated topics for need of advice included “*coping strategies”*, “*family, relationship and friends”, “fatigue”*, and *“body shape/sexuality”.*

The prevalence of elevated distress in AYA with cancer ranges between 12 and 41% (Dyson et al. 2012; Hedström et al. 2005; Kaul et al. 2017; Zabora et al. 2001; Zebrack et al. 2014). However, it is difficult to compare these results, because the studies vary in their methodology regarding the definition of AYA age, the point in time after diagnosis, and the instruments employed for measurement of distress.

Anxiety and depressive symptoms are frequently reported problems among AYA cancer patients (Sun et al. 2019). Due to disruptions in their developmental trajectory, they are at risk for depression (Park and Rosenstein 2015). The rates of depression and other psychological disorders are substantially higher in AYA with cancer than compared with older adults (Park and Rosenstein 2015). Recently, Giberson et al. reported a higher rate of suicidal ideation by AYAs versus older adults (Giberson et al. 2021). In the present study, 26% of participants reported experiencing depressive symptoms and 15% moderate or severe anxiety symptoms. This is in line with the results of an epidemiological study across all major tumor entities. In that study, 29.5% of the AYA complained about symptoms of depression, and 20.8% suffered from anxiety (Geue et al. 2018). In contrast, Giberson and colleagues observed 40.4% of AYA cancer survivors reporting depressive symptoms by applying the PHQ-9 questionnaire (Giberson et al. 2021). However, the definition of the cut-off score ≥ 5 was lower in comparison to our study with a cut-off ≥ 10, which may explain the different results.

The prevalence of fear of cancer recurrence in AYA cancer survivors ranges between 31% and 85.2%. Higher fear of cancer recurrence levels is associated with lower scores on levels of physical and psychological functioning as well as overall health-related QoL, higher treatment intensity, and psychological distress (Yang et al. 2019). The observed 36% reported in the current study fits within this range. In addition, fear of progression was significantly associated with having children, which was also reported by others (Mehnert et al. 2009). In our study, survivors with lower educational qualifications and patients using the Internet as source for information reported higher values of fear of cancer recurrence. Upon further analysis of AYA cancer survivor interview data, it can be seen that the Internet, while providing a positive influence in terms of empowerment, also provides negative influence in terms of fear and uncertainty about health and the future (Cheung et al. 2021).

This cross-sectional study is limited by a reliance on self-reports. Furthermore, as we recruited patients at a single academic medical center, the sample size of our study is relatively small, leading to limited representativeness. In addition, the probability of bias by non-response should be considered. Due to inconsistent utilization of age boundaries and variations in the methodology of age-specific research, it is sometimes difficult to compare results with previous studies. In addition, the utilized control group (Geue et al. 2014) refers to data collected in 1998 and 2012, which may jeopardize the comparability of results, since it precludes proper adjustment for potential confounding factors. Thus, the findings should be interpreted with caution and cannot be generalized.

## Conclusion

Empirical research in a representative and large cohort of young cancer survivors is essential to map the challenges of young adults with cancer in comparison to healthy peers. In the future, longitudinal studies to determine within-person changes over time and recruitment from multiple institutions within the German AYA-Network will lead to the collection of larger samples and the identification of risk groups for psychosocial sequelae. Since there is a need for age-appropriate support services, the results of longitudinal studies are a prerequisite to establish survivorship concepts.

Despite these limitations, our study provides further insight into the burden of psychosocial distress among young adult cancer survivors. The patients reported excessive demands were overburdening them in daily life situations and upon resumption of work. However, employment was associated with lower levels of psychosocial distress, fatigue, anxiety, and depression. The restoration of earning capacity is therefore of great importance. Psycho-oncological and social support are valuable resources regarding those issues and need to become an inherent part of the aftercare of survivors of cancer during young adulthood.

## Supplementary Information

Below is the link to the electronic supplementary material.

Supplementary file1 (DOCX 54 KB)

## Declarations

### Data availability

The datasets generated during and/or analyzed during the current study are available from the corresponding author on reasonable request.
