# Supplementary material for: Psychosocial distress in young adults surviving hematological malignancies: a pilot study
Source: J Cancer Res Clin Oncol. 2022 Dec 17;149(9):5655–63. doi: 10.1007/s00432-022-04527-8 (PMC10356626; doi:10.1007/s00432-022-04527-8)
Supplement: Supplementary file 1 — Supplementary file1 (DOCX 54 KB) [file 432_2022_4527_MOESM1_ESM.docx]

**Supplementary**

**Journal of Cancer Research and Clinical Oncology**

**Psychosocial distress in young adults surviving hematological malignancies**

Andreas Wittwer^1^, Kristin Sponholz^1,2^, Jochen J Frietsch^1,3^, Paul Linke^1^, Peter Kropp^4^, Andreas Hochhaus^1^, Inken Hilgendorf^1^

**Corresponding author:**

apl. Prof. Dr. med. Inken Hilgendorf

ORCID: 0000-0003-2038-9730

Universitätsklinikum Jena, Abteilung Hämatologie und Internistische Onkologie

Am Klinikum 1, 07747 Jena, Germany

Phone: +49 (0) 36 41 / 9-32 4664

Fax: +49 (0) 36 41 / 9-32 4237

inken.hilgendorf@med.uni-jena.de

**Table S1: Scales of the EORTC QLQ-C30, comparison of study population and a former published reference population**

|  | Total | | | Female | | male | |  |
| --- | --- | --- | --- | --- | --- | --- | --- | --- |
|  | YA | Ref. pop. | YA vs. Ref.-pop. | YA | Ref.-pop. | YA | Ref.-pop | female versus male |
|  | N=46  *N=45 | N=585 | p | N=27 | N=385 | N=19  *N=18 | N=200 | p |
| Quality of life* | | | | | | | | |
| Mean  SD | 73.3  (17.7) | 80.4  (18.3) | 0.013 | 75.9  (15.4) | 79.2  (18.7) | 69.4  (20.6) | 82.7  (17.5) | 0.234 |
| Function Scales: | | | | | | | | |
| Physical Function |  |  |  |  |  |  |  |  |
| Mean  SD | 80.0  (20.4) | 96.8  (9.8) | <0.001 | 82.2  (19.3) | 96.3  (10.7) | 76.4  (21.9) | 97.9  (7.6) | 0.384 |
| Role Function |  |  |  |  |  |  |  |  |
| Mean  SD | 71.4  (31.6) | 94.8  (16.3) | <0.001 | 75.9  (30.1) | 93.7  (17.9) | 64.9  (33.3) | 96.9  (12.6) | 0.248 |
| Emotional Function* |  |  |  |  |  |  |  |  |
| Mean  SD | 64.8  (30.7) | 82.1  (20.6) | <0.001 | 66.4  (33.5) | 80.7  (21.1) | 62.5  (26.7) | 84.9  (19.5) | 0.684 |
| Cognitive Function* |  |  |  |  |  |  |  |  |
| Mean  SD | 74.8  (26.7) | 95.4  (13.1) | <0.001 | 75.9  (25.5) | 94.6  (14.4) | 73.2  (29.2) | 96.8  (10.1) | 0.738 |
| Social Function* |  |  |  |  |  |  |  |  |
| Mean  SD | 67.4  (34.8) | 94.6  (15.5) | <0.001 | 71.6  (34.5) | 93.1  (17.4) | 61.1  (35.2) | 97.5  (10.5) | 0.328 |
| Symptom Scales: | | | | | | | | |
| Nausea/vomiting |  |  |  |  |  |  |  |  |
| Mean  SD | 6.9  (13.4) | 2.8  (10.9) | 0.048 | 7.4  (15.6) | 3.6  (12.2) | 6.1  (10.0) | 1.2  (7.75) | 0.756 |
| Pain* |  |  |  |  |  |  |  |  |
| Mean  SD | 27.0  (31.8) | 7.9  (18.5) | <0.001 | 24.7  (31.8) | 9.3  (20.0) | 30.6  (32.5) | 5.2  (15.0) | 0.551 |
| Fatigue |  |  |  |  |  |  |  |  |
| Mean  SD | 38.8  (30.9) | 11.3  (18.9) | <0.001 | 36.6  (30.3) | 13.3  (20.1) | 42.0  (32.3) | 7.4  (15.7) | 0.574 |
| Single items: | | | | | | | | |
| Dyspnea |  |  |  |  |  |  |  |  |
| Mean  SD | 21.0  (26.6) | 2.4  (10.6) | <0.001 | 12.3  (18.8) | 3.3  (12.3) | 33.3  (31.4) | 0.7  (5.8) | 0.007 |
| Insomnia |  |  |  |  |  |  |  |  |
| Mean  SD | 36.2  (35.0) | 7.2  (19.4) | <0.001 | 29.6  (31.1) | 8.1  (19.9) | 45.6  (38.8) | 5.5  (18.2) | 0.129 |
| Appetite loss |  |  |  |  |  |  |  |  |
| Mean  SD | 9.4  (20.7) | 3.9  (14.1) | 0.083 | 11.1  (22.6) | 5.3  (16.4) | 7.0  (17.8) | 1.2  (7.0) | 0.515 |
| Constipation |  |  |  |  |  |  |  |  |
| Mean  SD | 11.6  (21.3) | 1.5  (7.9) | 0.003 | 9.9  (20.3) | 2.0  (9.3) | 14.0  (23.1) | 0.5  (4.1) | 0.521 |
| Diarrhea* |  |  |  |  |  |  |  |  |
| Mean  SD | 14.1  (24.1) | 2.9  (12.0) | 0.003 | 11.1  (24.5) | 4.1  (14.4) | 18.5  (23.5) | 0.5  (4.1) | 0.318 |
| Financial difficulties* |  |  |  |  |  |  |  |  |
| Mean  SD | 23.0  (30.8) | 3.3  (14.3) | <0.001 | 19.8  (29.6) | 4.0  (15.7) | 27.8  (32.8) | 2.0  (10.9) | 0.399 |

YA: young adults, SD; standard deviation, Ref.-pop.: reference population (Geue et al. 2014)

**Table S2: Multiple regression model - Quality of Life** (dependent variable: QLQ-C30 QoL scale)

|  |  | | | | | | |
| --- | --- | --- | --- | --- | --- | --- | --- |
| Independent variable | *b* | SE | *Β* | *t* | *p* | 95% CI | |
|  |  |  |  |  |  | *lower* | *upper* |
| (constant) | 140,652 | 37,043 |  | 3,797 | <0.001 | 65,370 | 215,933 |
| gender (0=m, 1=f) | 7,713 | 5,738 | 0,214 | 1,344 | 0,188 | -3,947 | 19,373 |
| age (continuous variable) | -0,109 | 0,509 | -0,035 | -0,213 | 0,832 | -1,144 | 0,926 |
| BMI (continuous variable) | -0,211 | 0,468 | -0,071 | -0,451 | 0,655 | -1,161 | 0,739 |
| A-level or higher (0=y, 1=n) | 1,315 | 6,141 | 0,037 | 0,214 | 0,832 | -11,166 | 13,796 |
| in a relationship (0=y, 1=n) | -10,383 | 5,652 | -0,291 | -1,837 | 0,075 | -21,869 | 1,103 |
| children (0=y, 1=n) | -2,890 | 5,807 | -0,080 | -0,498 | 0,622 | -14,690 | 8,911 |
| employed (0=y, 1=n) | -27,859 | 7,000 | -0,635 | -3,980 | <0.001 | -42,084 | -13,634 |
| acute leukemia (0=y, 1=n) | 0,696 | 6,681 | 0,017 | 0,104 | 0,918 | -12,882 | 14,274 |
| SCT treatment (0=y, 1=n) | -12,338 | 6,904 | -0,335 | -1,787 | 0,083 | -26,370 | 1,693 |
| note: N = 44; R² = 0,414; adjusted R² = 0,259; F(9, 34) = 2,668; sign. 0,019 | | | | | | | |

**Table S3 : Multiple regression model - Fatigue** (dependent variable : QLQ-Qc30 Fatigue scale)

|  |  | | | | | | |
| --- | --- | --- | --- | --- | --- | --- | --- |
| Independent variable | *b* | SE | *Β* | *t* | *p* | 95% CI | |
|  |  |  |  |  |  | *lower* | *upper* |
| (constant) | -111,894 | 65,957 |  | -1,696 | 0,099 | -245,935 | 22,146 |
| gender (0=m, 1=f) | 1,632 | 10,216 | 0,026 | 0,160 | 0,874 | -19,129 | 22,394 |
| age (continuous variable) | 0,360 | 0,907 | 0,068 | 0,397 | 0,694 | -1,483 | 2,203 |
| BMI (continuous variable) | 1,191 | 0,833 | 0,231 | 1,431 | 0,162 | -0,500 | 2,883 |
| A-level or higher (0=y, 1=n) | 4,513 | 10,935 | 0,073 | 0,413 | 0,682 | -17,710 | 26,735 |
| in a relationship (0=y, 1=n) | 11,690 | 10,063 | 0,188 | 1,162 | 0,253 | -8,761 | 32,141 |
| children (0=y, 1=n) | 8,651 | 10,339 | 0,138 | 0,837 | 0,409 | -12,360 | 29,662 |
| employed (0=y, 1=n) | 41,191 | 12,463 | 0,539 | 3,305 | 0,002 | 15,863 | 66,520 |
| acute leukemia (0=y, 1=n) | 6,411 | 11,896 | 0,090 | 0,539 | 0,593 | -17,765 | 30,588 |
| SCT treatment (0=y, 1=n) | 3,466 | 12,294 | 0,054 | 0,282 | 0,780 | -21,518 | 28,450 |
| note: N = 44; R² = 0,388; adjusted R² = 0,226; F(9, 34) = 2,397; sign. 0,032 | | | | | | | |

**Table S4: Multiple regression model - Stress** (dependent variable PHQ-S score)

|  |  | | | | | | |
| --- | --- | --- | --- | --- | --- | --- | --- |
| Independent variable | *b* | SE | *Β* | *t* | *p* | 95% CI | |
|  |  |  |  |  |  | *lower* | *upper* |
| (constant) | -4,413 | 9,234 |  | -0,478 | 0,636 | -23,159 | 14,334 |
| gender (0=m, 1=f) | 0,258 | 1,427 | 0,030 | 0,181 | 0,857 | -2,638 | 3,154 |
| age (continuous variable) | -0,094 | 0,127 | -0,127 | -0,742 | 0,463 | -0,353 | 0,164 |
| BMI (continuous variable) | 0,072 | 0,117 | 0,100 | 0,616 | 0,542 | -0,165 | 0,309 |
| A-level or higher (0=y, 1=n) | 2,381 | 1,511 | 0,278 | 1,576 | 0,124 | -0,687 | 5,448 |
| in a relationship (0=y, 1=n) | 1,162 | 1,395 | 0,134 | 0,833 | 0,410 | -1,669 | 3,993 |
| children (0=y, 1=n) | -0,966 | 1,444 | -0,111 | -0,669 | 0,508 | -3,897 | 1,966 |
| employed (0=y, 1=n) | 3,957 | 1,735 | 0,369 | 2,280 | 0,029 | 0,434 | 7,479 |
| acute leukemia (0=y, 1=n) | 1,837 | 1,667 | 0,184 | 1,102 | 0,278 | -1,547 | 5,221 |
| SCT treatment (0=y, 1=n) | -0,366 | 1,724 | -0,041 | -0,212 | 0,833 | -3,866 | 3,134 |
| note: N = 45; R² = 0,372; adjusted R² = 0,21; F(9, 35) = 2,3; sign. 0,038 | | | | | | | |

**Table S5: Multiple regression model - Depression** (dependent variable PHQ-9 score)

|  |  | | | | | | |
| --- | --- | --- | --- | --- | --- | --- | --- |
| Independent variable | *b* | SE | *Β* | *t* | *p* | 95% CI | |
|  |  |  |  |  |  | *lower* | *upper* |
| (constant) | -24,526 | 12,357 |  | -1,985 | 0,055 | -49,613 | 0,560 |
| gender (0=m, 1=f) | 2,483 | 1,909 | 0,219 | 1,301 | 0,202 | -1,392 | 6,358 |
| age (continuous variable) | -0,031 | 0,170 | -0,032 | -0,184 | 0,855 | -0,377 | 0,314 |
| BMI (continuous variable) | 0,298 | 0,156 | 0,317 | 1,906 | 0,065 | -0,019 | 0,615 |
| A-level or higher (0=y, 1=n) | 1,838 | 2,022 | 0,164 | 0,909 | 0,370 | -2,267 | 5,943 |
| In a relationship (0=y, 1=n) | 1,008 | 1,866 | 0,089 | 0,540 | 0,592 | -2,780 | 4,797 |
| children (0=y, 1=n) | 2,313 | 1,932 | 0,204 | 1,197 | 0,239 | -1,610 | 6,236 |
| employed (0=y, 1=n) | 5,292 | 2,322 | 0,377 | 2,279 | 0,029 | 0,578 | 10,005 |
| Acute leukaemia (0=y, 1=n) | 3,273 | 2,230 | 0,251 | 1,467 | 0,151 | -1,255 | 7,801 |
| SCT treatment (0=y, 1=n) | -0,287 | 2,307 | -0,024 | -0,124 | 0,902 | -4,970 | 4,397 |
| note: N = 45; R² = 0,343; adjusted R² = 0,174; F(9, 35) = 2,033; sign. 0,065 | | | | | | | |

**Table S6: Multiple regression model - Anxiety** (dependent variable GAD-7 score)

|  |  | | | | | | |
| --- | --- | --- | --- | --- | --- | --- | --- |
| Independent variable | *b* | SE | *Β* | *t* | *p* | 95% CI | |
|  |  |  |  |  |  | *lower* | *upper* |
| (constant) | -16,005 | 9,924 |  | -1,613 | 0,116 | -36,152 | 4,142 |
| gender (0=m, 1=f) | 0,750 | 1,533 | 0,081 | 0,489 | 0,628 | -2,362 | 3,863 |
| age (continuous variable) | -0,011 | 0,137 | -0,014 | -0,081 | 0,936 | -0,289 | 0,266 |
| BMI (continuous variable) | 0,066 | 0,125 | 0,086 | 0,529 | 0,600 | -0,188 | 0,321 |
| A-level or higher (0=y, 1=n) | 3,159 | 1,624 | 0,344 | 1,946 | 0,060 | -0,137 | 6,456 |
| in a relationship (0=y, 1=n) | -0,312 | 1,499 | -0,034 | -0,208 | 0,836 | -3,354 | 2,731 |
| children (0=y, 1=n) | 2,036 | 1,552 | 0,219 | 1,312 | 0,198 | -1,114 | 5,187 |
| employed (0=y, 1=n) | 4,843 | 1,865 | 0,422 | 2,597 | 0,014 | 1,057 | 8,629 |
| acute leukemia (0=y, 1=n) | 2,463 | 1,791 | 0,230 | 1,375 | 0,178 | -1,174 | 6,099 |
| SCT treatment (0=y, 1=n) | 0,131 | 1,853 | 0,014 | 0,071 | 0,944 | -3,631 | 3,892 |
| note: N = 45; R² = 0,368; adjusted R² = 0,206; F(9, 35) = 2,269; sign. 0,04 | | | | | | | |

**Table S7: Multiple regression model - Fear of progression** (dependent variable PA-F-KF score)

|  |  | | | | | | |
| --- | --- | --- | --- | --- | --- | --- | --- |
| Independent variable | *b* | SE | *β* | *t* | *p* | 95% CI | |
|  |  |  |  |  |  | *lower* | *upper* |
| (constant) | 19,495 | 18,700 |  | 1,043 | 0,304 | -18,468 | 57,459 |
| gender (0=m, 1=f) | 5,377 | 2,889 | 0,263 | 1,861 | 0,071 | -0,488 | 11,242 |
| age (continuous variable) | -0,431 | 0,258 | -0,245 | -1,672 | 0,103 | -0,954 | 0,092 |
| BMI (continuous variable) | 0,192 | 0,236 | 0,113 | 0,813 | 0,422 | -0,288 | 0,672 |
| A-level or higher (0=y, 1=n) | 8,224 | 3,060 | 0,407 | 2,688 | 0,011 | 2,012 | 14,436 |
| In a relationship (0=y, 1=n) | 2,385 | 2,824 | 0,117 | 0,844 | 0,404 | -3,348 | 8,118 |
| children (0=y, 1=n) | -6,428 | 2,924 | -0,314 | -2,198 | 0,035 | -12,365 | -0,492 |
| employed (0=y, 1=n) | 5,772 | 3,514 | 0,229 | 1,643 | 0,109 | -1,361 | 12,906 |
| Acute leukaemia (0=y, 1=n) | 4,958 | 3,375 | 0,211 | 1,469 | 0,151 | -1,895 | 11,810 |
| SCT treatment (0=y, 1=n) | -4,590 | 3,491 | -0,217 | -1,315 | 0,197 | -11,678 | 2,497 |
| note: N = 45; R² = 0,537; adjusted R² = 0,418; F(9, 35) = 4,509; sign. <0,001 | | | | | | | |
